# Morroniside attenuates nucleus pulposus cell senescence to alleviate intervertebral disc degeneration *via* inhibiting ROS-Hippo-p53 pathway

**DOI:** 10.3389/fphar.2022.942435

**Published:** 2022-09-16

**Authors:** Chengcong Zhou, Sai Yao, Fangda Fu, Yishan Bian, Zhiguo Zhang, Huihao Zhang, Huan Luo, Yuying Ge, Yuying Chen, Weifeng Ji, Kun Tian, Ming Yue, Hongting Jin, Peijian Tong, Chengliang Wu, Hongfeng Ruan

**Affiliations:** ^1^ Institute of Orthopaedics and Traumatology, The First Affiliated Hospital of Zhejiang Chinese Medical University, Hangzhou, Zhejiang, China; ^2^ Department of Pharmacy, The Second Affiliated Hospital, School of Medicine, Zhejiang University, Hangzhou, Zhejiang, China; ^3^ The Fourth Clinical Medical College of Zhejiang Chinese Medical University, Hangzhou, Zhejiang, China; ^4^ Department of Orthopaedic, The First Affiliated Hospital of Zhejiang Chinese Medical University, Hangzhou, Zhejiang, China; ^5^ Department of Physiology, Zhejiang Chinese Medical University, Hangzhou, Zhejiang, China

**Keywords:** intervertebral disc degeneration, morroniside, nucleus pulposus, cell senescence, hippo signaling

## Abstract

Intervertebral disc (IVD) degeneration (IVDD) which is highly prevalent within the elderly population, is a leading cause of chronic low back pain and disability. Nucleus pulposus (NP) cell senescence plays an indispensable role in the pathogenesis of IVDD. Morroniside is a major iridoid glycoside and one of the quality control metrics of Cornus officinalis Siebold & Zucc (CO). An increasing body of evidence suggests that morroniside and CO-containing formulae share many similar biological effects, including anti-inflammatory, anti-oxidative, and anti-apoptotic properties. In a previous study, we reported that Liuwei Dihuang Decoction, a CO-containing formula, is effective for treating IVDD by targeting p53 expression; however, the therapeutic role of morroniside on IVDD remains obscure. In this study, we assessed the pharmacological effects of morroniside on NP cell senescence and IVDD pathogenesis using a lumbar spine instability surgery-induced mouse IVDD model and an *in vitro* H_2_O_2_-induced NP cell senescence model. Our results demonstrated that morroniside administration could significantly ameliorate mouse IVDD progression, concomitant with substantial improvement in extracellular matrix metabolism and histological grading score. Importantly, *in vivo* and *in vitro* experiments revealed that morroniside could significantly reduce the increase in SA-β-gal activities and the expression of p53 and p21, which are the most widely used indicators of senescence. Mechanistically, morroniside suppressed ROS-induced aberrant activation of Hippo signaling by inhibiting Mst1/2 and Lats1/2 phosphorylation and reversing Yap/Taz reduction, whereas blockade of Hippo signaling by Yap/Taz inhibitor-1 or Yap/Taz siRNAs could antagonize the anti-senescence effect of morroniside on H_2_O_2_-induced NP cell senescence model by increasing p53 expression and activity. Moreover, the inhibition of Hippo signaling in the IVD tissues by morroniside was further verified in mouse IVDD model. Taken together, our findings suggest that morroniside protects against NP cell senescence to alleviate IVDD progression by inhibiting the ROS-Hippo-p53 pathway, providing a potential novel therapeutic approach for IVDD.

## Introduction

Low back pain (LBP) is one of the most common health problems encountered clinically that affects up to 80% of individuals during their lifetime, leading to significant disability worldwide and imposing substantial socioeconomic and medical costs (Smith et al., 2011; Risbud and Shapiro, 2014; Qaseem et al., 2017). Intervertebral disc (IVD) degeneration (IVDD) refers to the pathophysiological process of natural degeneration and aging of IVD, which is clinically associated with LBP and recognized as the main pathogenic factor for LBP (Hoy et al., 2012). Although many risk factors have been identified, e.g., mechanical stress, inflammation, and aging, the exact molecular biological mechanisms which initiate and promote IVDD remain obscure. Notwithstanding that the current therapeutic approach, including medication, physical therapy, or surgical intervention, can relieve clinical symptoms, the underlying pathophysiological mechanisms underlying IVDD progression can not be targeted (Zhang et al., 2017a). Accordingly, further research is warranted to identify disease-modifying therapies.

IVD is a fibrous cartilaginous tissue composed of central aggrecan rich, highly hydrated gel-like nucleus pulposus (NP) surrounded by lamellated annulus fibrosus (AF); and cartilaginous endplates (CEP) interfacing with NP and AF at superior and inferior boundaries, connecting two adjacent vertebral bodies and transmitting mechanical loads applied to the spinal column. It has been well established that IVDD is a chronic process characterized by upregulation of Aggrecanases in the NP, such as A Disintegrin And Metalloproteinase with Thrombospondin 5 (Adamts-5), which is responsible for progressive Aggrecan degradation, leading to impaired disc function and accelerated degeneration (Liang et al., 2022). Emerging evidence from clinical and animal model studies demonstrates that senescent IVD cells, particularly NP cells, accumulate in aged and degenerated discs, as indicated by increased SA-β-gal-positive cells and activated p53-p21 pathway, which is recognized as a new hallmark and major cause of IVDD (Zhang et al., 2021a; Sun et al., 2021; Veroutis et al., 2021). Moreover, senescent cells secrete a series of proinflammatory cytokines and proteases, affecting the local environment in a paracrine manner, leading to senescence of peripheral cells and tissue dysfunction (Acosta et al., 2013). Therefore, targeting NP cell senescence represents an effective strategy to alleviate IVDD progression and has huge prospects for clinical application (Roberts et al., 2006; Le Maitre et al., 2007; Kim et al., 2008; He and Sharpless, 2017; Zhang et al., 2021a; Shao et al., 2021).

Hippo signaling is an evolutionarily conserved pathway in tissue homeostasis, organogenesis, and tumorigenesis that regulates cell senescence, cell proliferation, and apoptosis (Shao et al., 2014). In mammals, the core kinase cascade of canonical Hippo pathway involves mammalian sterile 20-like kinases 1 and 2 (Mst1/2) and two large tumor suppressors kinase 1 and 2 (Lats1/2). The Yes-associated protein (Yap) and its paralogue PDZ-binding motif (Taz) are downstream transducers of Hippo signaling, functioning as negative regulators of Hippo signaling. Once the Hippo signaling is activated, Mst1/2 phosphorylates and activates Lats1/2, which in turn phosphorylates Yap/Taz, resulting in their cytoplasmic retention and proteolytic degradation. When the Hippo pathway is inactive, unphosphorylated Yap/Taz translocates into the nucleus and interacts with TEA-domain (Tead) transcription family (Tead1-4) to promote the transcription of target genes, including *Cyr61*, *Ctgf,* and *Ankrd1* (Mo et al., 2014). Accruing works have shown that dysregulation of the Hippo pathway is involved in natural aging (Zhang et al., 2021b), excessive mechanical stress (Zhang et al., 2018a; Croft et al., 2021), or poly (methyl methacrylate) particles (Ge et al., 2019)-induced IVDD progression. Recent evidence from a tumor senescence study suggested that Taz, a core component and transcriptional coactivator of Hippo signaling, could negatively regulate p53 and attenuate p53-mediated cellular senescence (Miyajima et al., 2020), which repairs the NP senescent phenotype (Fearing et al., 2019) as well as osteoporosis and skeletal aging disease (Yu et al., 2018).

Morroniside (MR) is a major iridoid glycoside and key quality control metric of Cornus officinalis Siebold & Zucc (CO) that has been widely used for food and as a medicinal herb in Traditional Chinese Medicine (TCM). In recent decades, the pharmacology and phytochemistry of CO have been extensively investigated. It has been established that the CO-containing formulae and MR have similar biological effects, including anti-inflammatory (Yu and Wang, 2018; Yu et al., 2021), anti-oxidative stress (Xu et al., 2006), and anti-apoptotic properties, to mitigate degenerative diseases (Pi et al., 2017; Yu et al., 2021). In a previous study, we found that MR could effectively attenuate cartilage degeneration and arthritic development (Yu et al., 2021), comparable to the therapeutic effect of CO-composed TCM formulas (Zhang et al., 2017b; Wang et al., 2018a; Dong et al., 2018; Yang et al., 2019; Zhang et al., 2019). Furthermore, we recently demonstrated that the classic TCM formula Liuwei Dihuang Decoction (a CO-containing formula) could effectively impede IVDD progression by reversing the high expression of p53 protein in NP cells (Zhang et al., 2021c). Based on the above results, we hypothesize that MR yields a similar effect on p53-mediated NP cell senescence and IVDD development.

Herein, we investigated the therapeutic effect of MR on lumbar spine instability (LSI) surgery-induced mouse IVDD model and H_2_O_2_-induced NP cell senescence model. Our findings highlighted that MR could be a promising therapeutic agent in the treatment of IVDD.

## Materials and methods

### Reagents and antibodies

MR powder (99.67% purity) was provided by Manster TCM Co., Ltd., (Sichuan, China) and was authenticated by the authors (Suplementary Figure S1A). A voucher specimen (No. MUST-17071001) has been deposited at the First Affiliated Hospital of Zhejiang Chinese Medical University. Isoflurane was acquired from RWD Corp. (Shenzhen, China). Primary antibodies against Aggrecan, Adamts-5, p53, p21, and p16 were purchased from Ruiying Biological Co., (Jiangsu, China). Primary antibodies against Mst1/2, Phospho-Mst1 (Thr183)/Mst2 (Thr180) (p-Mst1/2), Lats1/2, phospho-Lats1/2 (Thr1079/1041) (p-Lats1/2), and Yap were obtained from Cell Signaling Technology (Beverly, MA, United States). Primary antibodies against Taz, Tead1, and Tead4 were supplied by Abcam Company Ltd., (Cambridge, MA, United States). Primary antibodies against Tead2 and Tead3 were from CUSABIO (Houston, TX, United States). GAPDH antibody was provided by OriGene (Rockville, MD, United States). IRDye 680LT and IRDye 800CW secondary antibodies were from Li-COR Biosciences (Lincoln, NE, United States). Fetal bovine serum (FBS, VS.500T, Australian origin) was ordered from Ausbian (Shanghai, China). Yap/Taz inhibitor-1 was purchased from MedChemExpress (Shanghai, China). DCFH-DA kit (E004-1-1) was purchased from Nanjing Jiancheng Bioengineering Institute (Nanjing, China). The small interfering RNAs (siRNAs) targeting *Yap* and *Taz* were chemically synthesized by GenePharma Co., (Shanghai, China). Unless otherwise mentioned, all chemicals were supplied by Sigma-Aldrich (St. Louis, MO, United States).

### Animals and treatments

Forty adult male C57BL/6J mice (8-week-old, 22 ± 2 g) were obtained from the animal experiments center of Zhejiang Chinese Medical University (Grade SPF, SCXK (Shanghai): 2017-0005). All mice were maintained within a specific pathogen-free animal care facility and housed in a room at 23°C ± 2°C, with a 12–12 h light/dark cycle, and mice had *ad libitum* access to water and lab chow. All experimental protocols were approved by the Ethics Committee on Animal Experimentation of Zhejiang Chinese Medical University (NO. IACUC-20190930-03).

All mice were randomly divided into four groups (*n* = 10 per group): Sham group, Model group, MR-L group, and MR-H group. All mice except those in the Sham group underwent IVDD modeling. The LSI surgery-induced mouse IVDD model was established as previously described (Fu et al., 2021). Briefly, mice were anesthetized with isoflurane and placed on the surgical table in a prone position. A longitudinal incision was created along the dorsal midline and the posterior paravertebral muscles adjacent to the L3∼L5 vertebrae were separated to expose the lower lumbar spine. The spinous process of L3∼L5 segments, supraspinous and interspinous ligaments were resected. The Sham group (placebo surgery) was a Sham surgical intervention that omitted the critical steps for IVDD modeling. Thus, mice in the Sham group only underwent a separation of the posterior paravertebral muscles from the L3∼L5 vertebrae. Finally, incisions were sutured and gentamicin hydrochloride was used to prevent wound infection.

From day 3 post-LSI surgery, mice in the MR-L and MR-H groups were intraperitoneally injected with MR (20 and 100 mg/kg body weight, respectively, dissolved in normal saline) 5 times a week, while mice in the Sham group and Model groups received an equal volume of normal saline. All mice were euthanized 8 weeks after LSI surgery and the corresponding lumbar vertebrae were harvested for further analysis.

### Micro-CT analysis

Before histological processing, the lumbar vertebrae were scanned by high-resolution micro-CT (Skyscan1176, Bruker micro-CT N.V., Kontich, Belgium) at a voltage of 50 kV with a current of 500 μA and a resolution of 9 μm per pixel. Image reconstruction and quantitative morphologic analysis were performed with NRecon v1.6 and CTAn v1.15 software, respectively. Three-dimensional (3D) images were presented by 3D model visualization software, CTVol v2.2. Coronal images of L3∼L4 vertebrae were selected for 3D histomorphometric analyses. Intervertebral disc height (DHI) was calculated by averaging the anterior, middle, and posterior distances of L3∼L4 IVD and comparing them to the average height of the adjacent upper and lower vertebral body.

### Histological staining, immunohistochemistry, and immunofluorescence analysis

All lumbar tissues were cut into 4 μm-section for H&E and Safranin O/Fast green staining. The histological score was graded by a blind pathologist, as previously described (Zhang et al., 2021c). For IHC and IF assay, sections were blocked with 5% normal goat serum for 1 h at room temperature, followed by incubation with primary antibodies Aggrecan (diluted 1:300), Adamts-5 (diluted 1:100), p53 (diluted 1:500), p21 (diluted 1:500), p16 (diluted 1:500), p-Mst1/2 (diluted 1:200), Mst1/2 (diluted 1:200), Taz (diluted 1:200), Ctgf (diluted 1:200), Tead1 (diluted 1:500), Tead2 (diluted 1:1,000), Tead3 (diluted 1:1,000), and Tead4 (diluted 1:500) at 4°C overnight, respectively. Negative control sections were incubated with nonspecific IgG. For IHC staining, a horseradish peroxidase streptavidin detection system (ZSGB-BIO, Beijing, China) was subsequently used to detect immunoactivity. For IF analysis, sections were incubated with fluorescent secondary-antibody for 30 min in the dark. Each experiment was repeated in triplicates. Quantitative histomorphometric analysis was conducted in a blinded manner with Image-Pro Plus Software version 6.0 (Media Cybernetics Inc., Rockville, Maryland, United States).

### Measurement of the activity of senescence-associated β-galactosidase

The activity of SA-β-gal was determined using an SA-β-gal Staining Kit (Beyotime Biotechnology, Shanghai, China), according to the manufacturer’s instructions. Briefly, cells and tissues were fixed with 4% paraformaldehyde for 15 min, incubated with a working solution containing 0.05 mg/ml 5-Bromo-4-chloro-3-indolyl-β-D-galactopyranoside (X-gal) at 37°C overnight. SA-β-gal-positive cells (blue color) were photographed and counted under a microscope (Carl Zeiss, Gottingen, Germany). Each experiment was performed in triplicates.

### Cell culture and treatments

Rat NP (rNP) cell lines were kindly gifted by Prof. Di Chen from Rush University Medical Center (Chicago, IL, United States) and cultured in DMEM (Invitrogen, Carlsbad, CA, United States) supplemented with 15% fetal bovine serum (FBS, Ausbian, Australia), 2 mM L-glutamine (Invitrogen), 0.1 mM nonessential amino acids (Invitrogen) in a humidified 5% CO_2_ atmosphere at 37°C (Oh et al., 2016). The medium was changed every 2 days.

### Cell viability assay

The CCK-8 assay was used to evaluate cell viability. NP cells were seeded into a 96-well plate at a density of 2000 cells/well for 24 h, treated with MR for 2 h, and then stimulated with H_2_O_2_ for another 2 h to induce senescence (*n* = 5). Twenty-four hours after H_2_O_2_ treatment, 100 μl DMEM with 10 μl CCK-8 solution was added to each well and incubated at 37°C for another 1 h. The absorbance was measured using a microplate reader (ELx808, Biotek Instruments, Winooski, VT, United States) at a wavelength of 450 nm.

### RNA isolation and real-time PCR

Total RNA was extracted from rNP cells using Trizol Reagent (Invitrogen). The concentration of RNA was determined by NanoDrop 2000 (Thermo Fisher Scientific, United States). An equal RNA amount (1 μg) was transcribed into cDNA using PrimeScript™ RT reagent Kit with gDNA Eraser (TaKaRa, Dalian, China). Gene expression was analyzed on QuantStudio™ 7 Flex qPCR system (Thermo Fisher Scientific, United States) as we previously described (Hu et al., 2021). The specific primers used are shown in Table 1. The relative mRNA levels of target genes were normalized to the housekeeping gene β-actin. The expression difference of each target gene was calculated by the 2^−∆∆Ct^ method (Hu et al., 2021). All experiments were conducted in triplicates.

**TABLE 1:** Primers used for quantitative RT-PCR.

Genes	Primer sequence (5′→3′)	Products (bp)
*β-actin*	F: TCG​TGC​GTG​ACA​TTA​AAG​AG	134
	R: ATT​GCC​GAT​AGT​GAT​GAC​CT	
*p53*	F: TGC​TGA​GTA​TCT​GGA​CGA​CA	225
	R: CAGGCACAAACACGAACC	
*p21*	F: TGC​TGA​GTA​TCT​GGA​CGA​CA	137
	R: CAGGCACAAACACGAACC	
*p16*	F: AGC​AGC​ATG​GAG​TCC​TCT​G	124
	R: GGG​TAC​GAC​CGA​AAG​TGT​T	
*Ankrd1*	F: AAAATCAGTGCCCGAGAC	197
	R: GCACCGAAGGTCATCAAG	
*Ctgf*	F: CCCGAGAAGGGTCAAGC	200
	R: TGCCCATCCCACAGGTC	
*Cyr61*	F: TGT​CTT​TGG​CAC​GGA​ACC​T	278
	R: CTG​CAT​AAG​TAA​ATC​GGA​CTG​G	

### Western blot analysis

Total protein of rNP cells seeded in a 6 cm dish was extracted using RIPA lysis buffer (Beyotime, Shanghai, China) containing 1% protease inhibitors for 30 min at 4°C. The protein concentration was determined using a Pierce™ BCA Protein Assay Kit (Thermo Scientific, Waltham, MA). An equal amount of protein (30 μg) was separated by SDS-PAGE and transferred onto nitrocellulose filter membranes (Millipore, Billerica, City, State, United States). After blocking with 5% non-fat milk for 1 h, membranes were respectively incubated with primary antibodies against p53 (1:1,000), p21 (1:500), p-Mst1/2 (1:1,000), Mst1/2 (1:1,000), p-Lats1/2 (1:1,000), Lats1/2 (1:1,000), p-Yap (1:1,000), Yap (1:1,000), Taz (1:1,000), GAPDH (1:1,000) at 4°C overnight. Then, IRDye 680 and 800 secondary antibodies (LI-COR, Lincoln, NE, United States) were added and incubated for 1 h. The signals were visualized with Odyssey Infrared Imaging System (LI-COR). Immunoreactive bands were quantified by Quantity ONE software (Bio-Rad, Hercules, CA, United States). GAPDH was used as the internal standard of total target proteins, and phosphorylated proteins were normalized to their corresponding total protein.

### Luciferase reporter assays for p53 protein

To examine the transcriptional activity of p53 protein, rNP cells were seeded in 24-well culture plates and transfected with 500 ng pp53-TA-luc-CP (Beyotime) and 20 ng pRL-null expressing Renilla luciferase (Promega, Madison, WI) using X-treme GENE HP DNA Transfection Reagent (Roche, Germany) for 24 h. Then, cells were treated with MR (200 μM), Yap/Taz inhibitor-1 (1 μM) as described in Figures 3D, 4F for another 24 h. Cell lysates were prepared in lysis buffer, and supernatants were used for dual-luciferase assay according to the manufacturer’s instructions (Promega). Each experiment was conducted in triplicates.

### Measurement of intracellular ROS levels

Intracellular generation of ROS was examined using a DCFH-DA kit, according to the manufacturer’s instructions. Briefly, rNP cells were inoculated into a 6 cm plate and treated with 200 or 400 μM MR for 2 h, followed by 200 μM H_2_O_2_ for 30 min. DCFH-DA was added and incubated for another 30 min at 37°C in the dark. DCFH-DA was oxidized by ROS to obtain the fluorescent product DCF. After rinsing with a serum-free culture medium, the fluorescence intensity of DCF was quantified with a microplate reader (Bio-TEK, Winooski, VT, United States) at an emission wavelength of 525 nm and an excitation wavelength of 500 nm.

### Yap and Taz small interfering RNAs transfection

Transfection of siRNA was performed on rNP cells using Lipofectamine RNAiMAX (ThermoFisher Scientific, MA, United States) according to the manufacturer’s instructions. A total of 2 × 10^5^ rNP cells were seeded in a 6-well plate for 24 h and then transfected with 5 μL Yap, Taz siRNAs or Negative Control (NC) siRNA (20 μM) (siRNA sequences were shown in Table 2) and 10 μL RNAiMAX. Then, cells were treated with or without H_2_O_2_ and MR (200 μM) after 24 h of transfection.

**TABLE 2:** siRNA sequences for targeting Yap and Taz.

Gene	siRNA sequence (5′ ê 3′)
siNC	GCGAC GAUCU GCCUA AGAU
si*Yap* 1#	UCUUC UGGUC AGAGA UACUU CUUAA
si*Yap* 2#	CCAAU AGUUC AGAUC CCUUU CUUAA
si*Taz* 1#	GUGAU GAAUC AGCCU CUGAA U
si*Taz* 2#	GCUCA UGAGU GUGCC CAAU

### Statistical analysis

All numerical data were presented as means ± SD. The statistical analysis was performed using GraphPad Prism software (San Diego, CA, United States). One-way analysis of variance test was performed to compare the means among groups, and multiple comparisons were performed using the least significant difference (LSD) test. A *p*-value less than 0.05 was statistically significant.

## Results

### Morroniside delays intervertebral disc degeneration development in lumbar spine instability surgery-induced intervertebral disc degeneration mice

**FIGURE 1:**
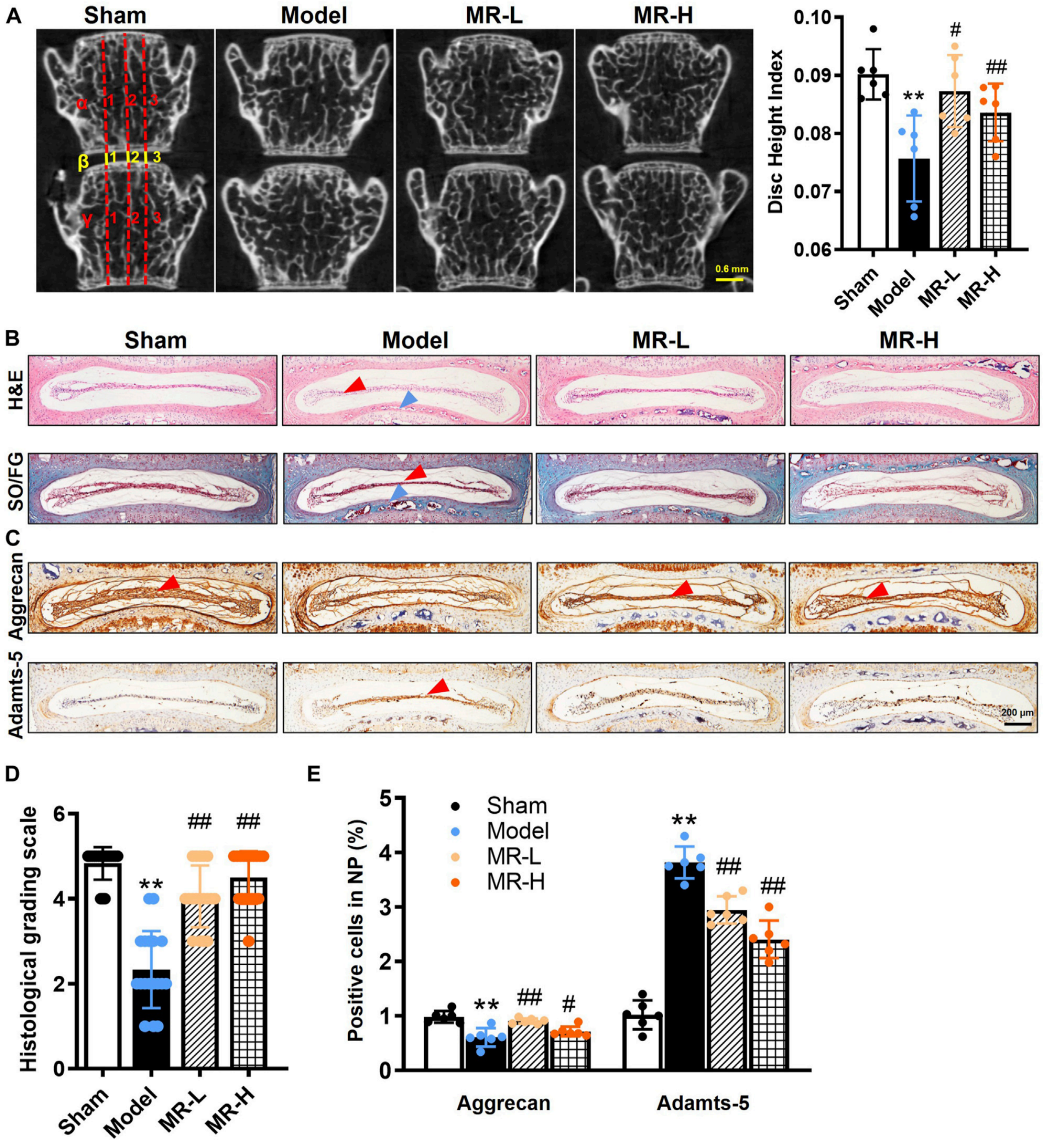
MR delays IVDD progression in LSI surgery-induced IVDD mice. **(A)** Representative micro-CT image of the lumbar spine. From day 3 post-LSI surgery, mice in the MR-L and MR-H groups were intraperitoneally injected with MR (20 and 100 mg/kg body weight, respectively) 5 times a week for 8 weeks. The DHI between the L3 and L4 vertebrae was calculated based on measurements of adjacent L3 and L4 vertebrae. Yellow lines represent the distances (β1-β3) between the adjacent vertebra, and red lines represent adjacent vertebral body heights (α1-α3 and γ1-γ3). DHI was calculated using the following equation: DHI = 2 (β1 + β2 + β3)/(α1 + α2 + α3 + γ1 + γ2 + γ3). **(B)** H&E staining and Safranin O/Fast green staining results of IVDs. Red arrowheads indicate flattened NP tissues, blue arrowheads indicate CEP. **(C)** The IHC staining results of Aggrecan and Adamts-5 in IVDs. Red arrowheads indicate positive staining cells. **(D)** Evaluation of IVDD degree by histological grading score. **(E)** Quantification of Aggrecan and Adamts-5 in **(C)**. Data are expressed as mean ± SD, ^**^ *p <* 0.01 (vs. Sham group); ^#^ *p <* 0.05, ^##^ *p <* 0.01 (vs. IVDD group), *n* = 10 per group.

To investigate the pharmacological effect of MR on IVDD development, mice that underwent LSI surgery were intraperitoneally injected with two optimized concentrations of MR (20 and 100 mg/kg, respectively) for 8 weeks. The radiographic changes observed in L3∼L4 IVD were determined by micro-CT analysis. Consistent with our previous findings (Fu et al., 2021), disc heights in IVDD mice were significantly decreased by roughly 20% compared to Sham mice. MR treatment reversed the decline in IVD height of IVDD mice (Figure 1A). To determine whether MR could affect the structure and composition of IVDs in IVDD mice, we examined histological changes in L3∼L4 IVD by H&E and Safranin O/Fast green staining. Consistent with micro-CT results, we found that IVDD mice exhibited ruptured IVDs with flattened NP, fissures and folds in outer AF, and heterotopic ossification in CEP. MR could block these structural and compositional impairments to a certain extent (Figure 1B). The degeneration of IVD was further assessed by the histological score system as previously described (Norcross et al., 2003). We found that MR restored the IVD score in IVDD mice by 70% to levels comparable to those observed in Sham mice (Figure 1D).

Subsequently, to evaluate the effects of MR on matrix metabolism of IVDD mice, the expressions of Aggrecan, a major extracellular matrix protein in NP tissue, and corresponding Aggrecanase, Adamts-5, were analyzed by IHC analysis. The results showed that MR could significantly alleviate the decrease in Aggrecan and increase in Adamts-5 in IVDD mice (Figures 1C,E). Our findings indicate that MR could attenuate LSI surgery-induced matrix degradation in NP tissues and IVDD progression.

### Morroniside reverses the nucleus pulposus cell senescent phenotype in intervertebral disc degeneration development mice

**FIGURE 2:**
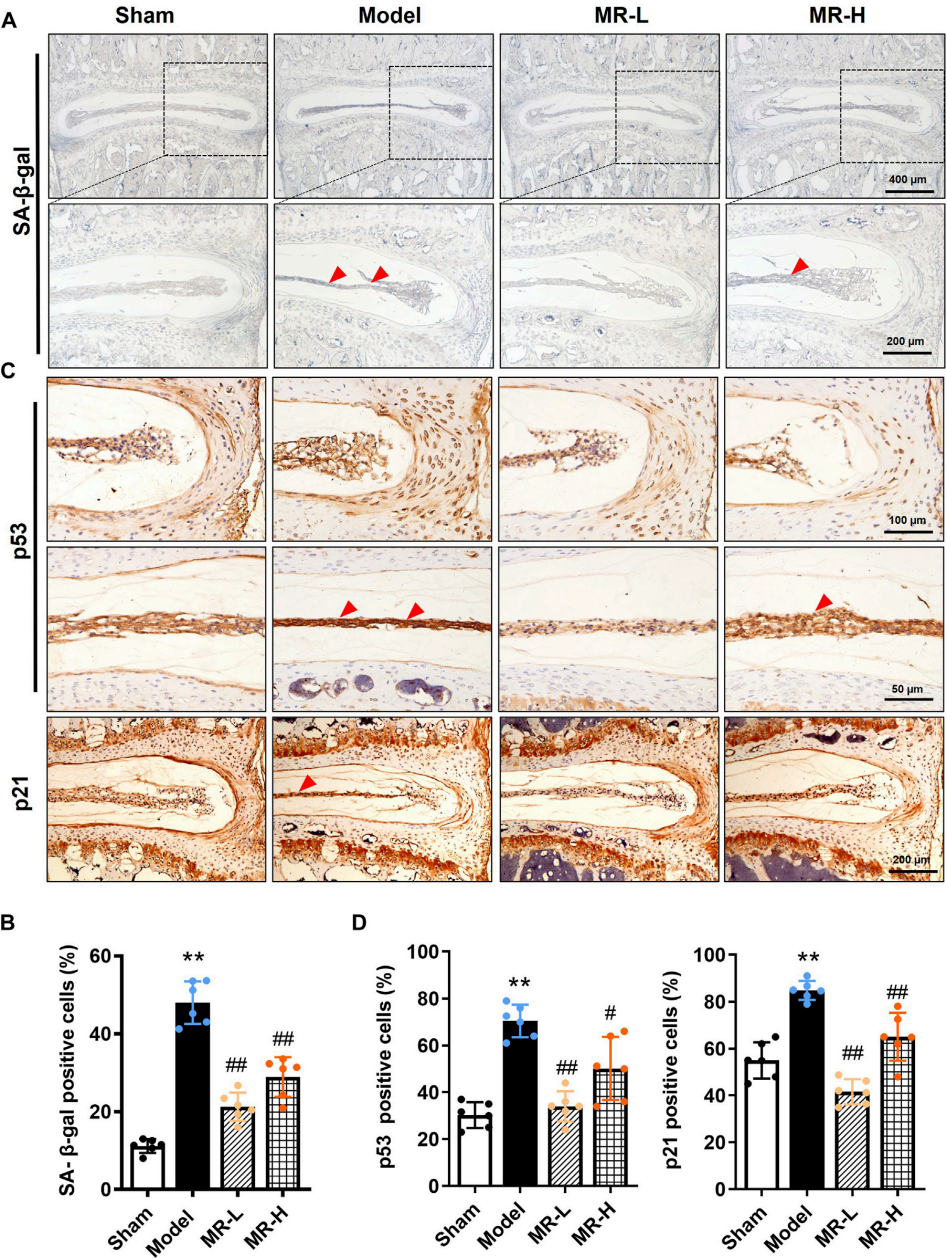
MR reverses the senescent phenotype of NP chondrocytes in IVDD mice. **(A)** The SA-β-gal staining results of IVDs of mice. Red arrowheads indicate positively-stained cells. **(B)** The ratio of SA-β-gal staining in **(A)**. **(C)** The IHC staining results of p53 and p21 in NP tissues of mice. Red arrowheads indicate positively-stained cells. **(D)** Quantification of p53-and p21-positive cells in NP tissues of mice in **(C)**. Data are expressed as the mean ± SD. ^**^ *p <* 0.01 (vs. Sham group); ^#^ *p <* 0.05, ^##^ *p <* 0.01 (vs. IVDD group), *n* = 10 per group.

Senescence is characterized by aberrant lysosomal activity detected by the SA-β-gal assay, and increased expression of p53, p21, and p16 proteins (He and Sharpless, 2017). An increasing body of evidence suggests that NP cell senescence is responsible for IVDD progression (Kim et al., 2009; Jeong et al., 2014; Dimozi et al., 2015). To assess whether MR affects the NP cell senescent phenotype in IVDD mice, we quantified SA-β-gal activity and the expression of p53, p21, and p16 in IVDs. We found that the average positive rate of SA-β-gal in NP tissues of IVDD mice was 4.5-fold higher than in Sham mice, whereas MR treatment reduced its proportion by approximately 50% (Figures 2A,B). Similarly, IHC analysis of p53 and p21 expression in IVDs indicated that MR impaired the elevated expression of p53 and p21 proteins in IVDD mice, particularly in NP tissues (Figures 2C,D). Intriguingly, no p16-positive cells were detected in IVD tissues (data not shown). The above results suggest that MR could suppress the NP cell senescent phenotype in IVDD mice.

### Morroniside inhibits cell senescence of nucleus pulposus cells induced by H_2_O_2_

To better understand the pharmacological molecular events underlying the inhibitory effect of MR on NP cell senescence, rNP cells were incubated with different concentrations of H_2_O_2_ to induce a senescence model as previously described (Du et al., 2022). As shown in Supplementary Figures S2A–C, the H_2_O_2_-induced rNP cell senescence model was confirmed by increased mRNA and protein expression of p53 and p21 and increased SA-β-gal-positive staining rate in rNP cells following H_2_O_2_ treatment. The H_2_O_2_ concentration was chosen based on the CCK-8 assay and the IC_50_ concentration (200 μM) used for all the following experiments (Supplementary Figure S3A).

**FIGURE 3:**
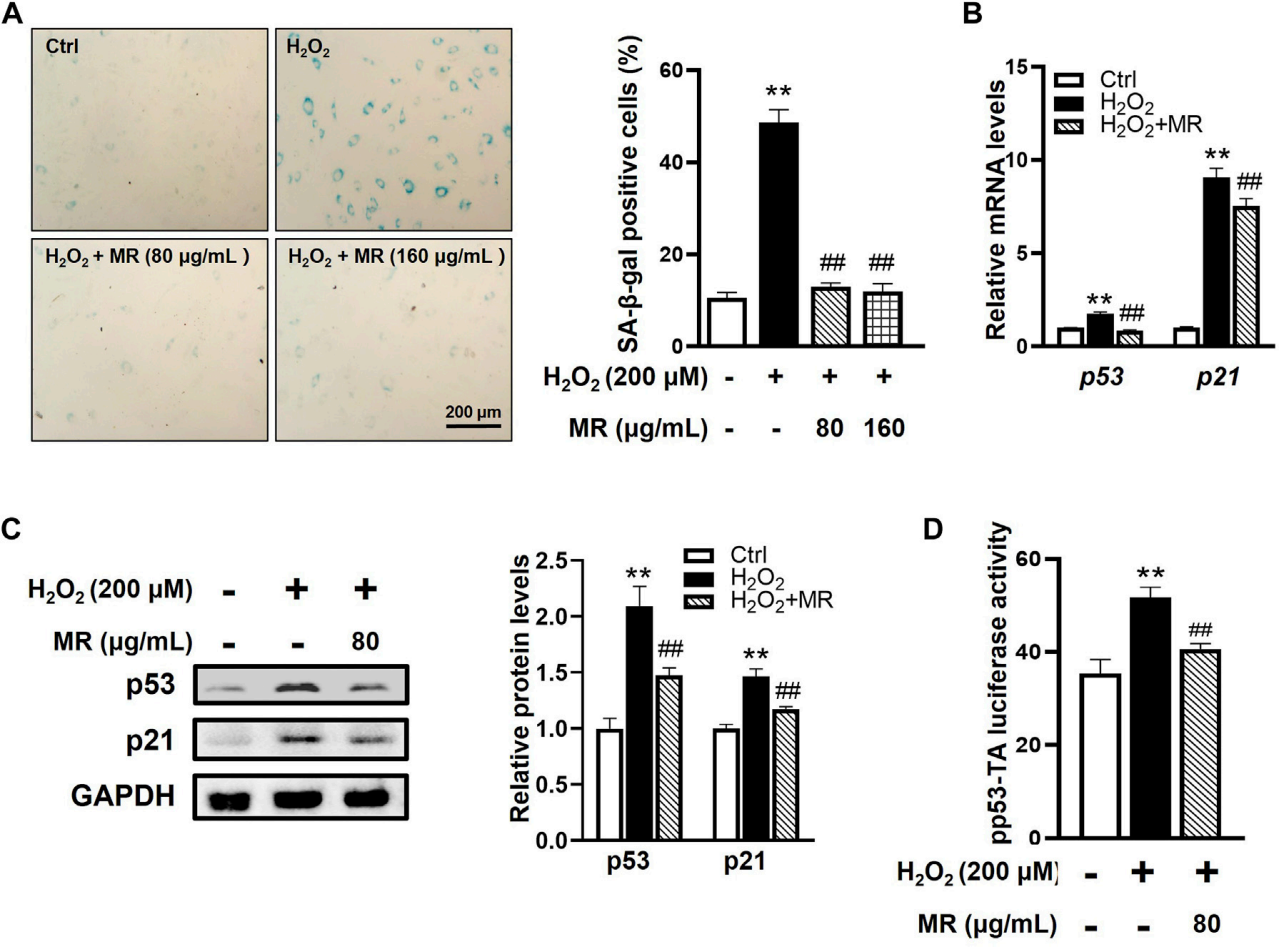
MR inhibits H_2_O_2_-induced cell senescence of rNP cells. rNP cells were pretreated with indicated concentrations (200 and 400 μM) of MR for 2 h and then treated with 200 μM H_2_O_2_ for another 2 h. **(A)** SA-β-gal staining results showed that MR treatment resulted in a reduction of SA-β-gal-positive cells in rNP cells induced by H_2_O_2_. **(B)** qPCR assay results showed that the expression of p53 and p21 mRNA induced by H_2_O_2_ was significantly decreased in MR-treated rNP cells. **(C)** Western blot results showed that MR supplementation reversed the enhanced expression of p53 and p21 proteins in H_2_O_2_-induced rNP cells. **(D)** rNP cells were transiently transfected with a pp53-TA-luc-CP reporter plasmid and cultured with or without H_2_O_2_ (200 μM) or MR (200 μM). Total cell lysates were subjected to luciferase assay. Data are expressed as the mean ± SD. **p < 0.01 compared with Control group; #p < 0.05, ##p < 0.01 compared to H_2_O_2_-treated group.

To identify whether MR could alleviate rNP cell senescent phenotype *in vitro*, rNP cells were pretreated with different concentrations of MR (200 and 400 μM, dissolved in sterilized water, respectively) for 2 h before H_2_O_2_ exposure. The CCK-8 assay results showed that both 200 and 400 μM MR could significantly improve the H_2_O_2_-impaired viability of rNP cells (Supplementary Figure S3A). In line with our *in vivo* findings, SA-β-gal staining revealed that MR caused a 36% decrease in the SA-β-gal-positive ratio in H_2_O_2_-treated rNP cells. Moreover, both concentrations of MR yielded a similar protective effect (Figure 3A). Thus, we selected 200 μM MR for subsequent *in vitro* experiments. As expected, MR also downregulated the mRNA and protein levels of p53 and p21 (Figures 3B,C). Subsequently, to further investigate the potential role of MR on p53 transcriptional activity, we transfected rNP cells with a pp53-TA-luc reporter plasmid. Of note, MR reduced the transcriptional activity of p53 to 80% of that induced by H_2_O_2_ alone (Figure 3D). Taken together, these results substantiate that MR protects against H_2_O_2_-induced NP cell senescence by inhibiting the p53-p21 pathway.

### Morroniside inhibits H_2_O_2_-induced ROS and hippo signaling activation in rat nucleus pulposus cells

Overwhelming evidence substantiates that excessive ROS-induced activation of Hippo signaling plays a vital role in NP cell senescence and IVDD development (Loforese et al., 2017; Zhang et al., 2018a; Du et al., 2022). Next, to elucidate the molecular mechanism underlying the protective effect of MR against H_2_O_2_-induced NP cell senescence, we examined the Hippo signaling activity of rNP cells after H_2_O_2_ exposure. As expected, western blot showed H_2_O_2_ could time-dependently induce phosphorylation of Mst1/2, Lats1/2, Yap, and Taz proteins and downregulate reduction of Yap and Taz expression in rNP cells (Supplementary Figures S2D,E). Consistent with these results, qPCR analysis of Hippo signaling target genes (*Ctgf*, *Cyr61*, *Ankrd1*) showed that the mRNA levels of *Ctgf*, *Cyr61*, and *Ankrd1* were significantly downregulated in H_2_O_2_-treated rNP cells (Supplementary Figure S2F). Furthermore, we found that H_2_O_2_ treatment significantly increased ROS levels in rNP cells (Supplementary Figure S2G). These findings suggest that H_2_O_2_ could activate ROS-mediated activation of Hippo signaling in rNP cells.

**FIGURE 4:**
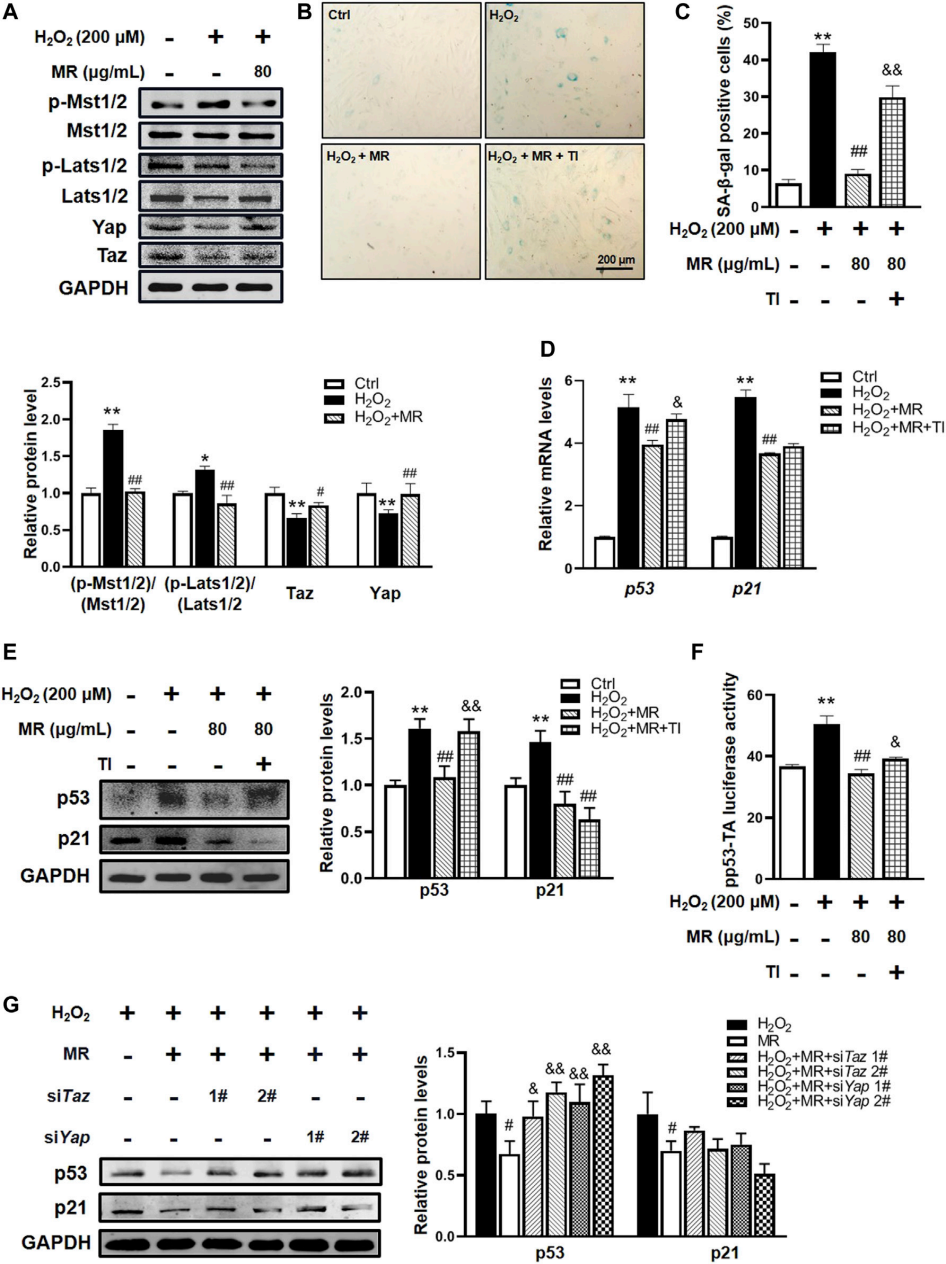
Hippo signaling participates in MR-mediated protection against H_2_O_2_-induced NP cell senescence. rNP cells were pretreated with the indicated concentration of MR (200 μM) for 2 h, followed by 200 μM H_2_O_2_ for another 2 h. **(A)** Western blot analysis results. **(B,C)** SA-β-gal staining analysis. rNP cells were pretreated with 1 μM Yap/Taz inhibitor-1 (YTI) and 200 μM MR for 2 h and then treated with 200 μM H_2_O_2_ for 2 h. **(D)** qPCR assay was applied to detect the expression of p53 and p21 mRNA levels in rNP cells. **(E)** Western blot results of p53 protein expression in response to 1 μM YTI treatment. **(F)** p53 luciferase reporter gene activity results. rNP cells were transiently transfected with a pp53-TA-luc-CP reporter plasmid and cultured with or without H_2_O_2_ (200 μM) or MR (200 μM) or 1 μM YTI for 48 h. Total cell lysates were subjected to luciferase assay. **(G)** Western blot results of p53 and p21 protein expressions in response to 200 μM MR with siRNA-mediated knockdown of Taz or Yap. rNP cells were treated with or without H_2_O_2_ and MR (200 μM) after 24 h of siRNA transfection. Data are representative of 3 independent repeat experiments. Values are expressed as mean ± SD. *p < 0.05, **p < 0.01 compared with control group. #p < 0.05, ##p < 0.01 compared with H_2_O_2_-treated cells. &p < 0.05, &&p < 0.01 compared with MR- and H_2_O_2_-treated cells.

To investigate whether MR affects Hippo signaling pathway in H_2_O_2_-treated rNP cells, we examined the phosphorylation status of Mst1/2, Lats1/2, and the total Yap and Taz levels in rNP cells. We found that MR disrupted H_2_O_2_-induced activated phosphorylation of Mst1/2 and Lats1/2 in rNP cells and overturned H_2_O_2_-induced reduction of Yap and Taz (Figure 4A,A′). Moreover, MR treatment significantly diminished H_2_O_2_-induced ROS increase (Supplementary Figure S2G). Our data indicate that MR might protect against H_2_O_2_-induced NP cell senescence by blocking the ROS-Hippo signaling pathway.

### Loss of function of Yap/Taz suppresses the protective effect of morroniside against H_2_O_2_-induced nucleus pulposus cell senescence

To further confirm the role of Hippo signaling on the possible mechanisms underlying the protective effects of MR on rNP cell senescence, Yap/Taz inhibitor-1, a selective Yap and Taz inhibitor, was added to rNP cells. Interestingly, SA-β-gal staining showed that Yap/Taz inhibitor-1 significantly abolished the effect of MR on the improvement of percentages of SA-β-gal-positive cells in the H_2_O_2_-treated rNP cell senescent model (Figures 4B,C). Unexpectedly but intriguingly, Yap/Taz inhibitor-1 countered the reduction of p53 mRNA and protein expression induced by MR but failed to alter p21 mRNA and protein expression (Figures 4D,E). Furthermore, the luciferase assay of p53 protein transcription activity showed that the Yap/Taz inhibitor-1 could block approximately 20% of MR-mediated inhibition of p53 activity (Figure 4F).

To reconfirm the role of Hippo signaling therein, we used siRNAs to specifically knockdown *Yap* and *Taz* expression in rNP cells. Western blot results showed that siRNAs targeting *Yap* and *Taz* could effectively reduce the expressions of Yap and Taz in rNP cells (Supplementary Figure S2H). Moreover, knockdown *Yap* and *Taz* significantly overturned the inhibition of MR on H_2_O_2_-induced p53 protein (Figure 4G). Taken together, the above results demonstrate that MR could attenuate H_2_O_2_-induced NP cell senescence *via* suppressing ROS-Hippo signaling.

### Morroniside inhibits hippo signaling in nucleus pulposus tissue of intervertebral disc degeneration development mice

**FIGURE 5:**
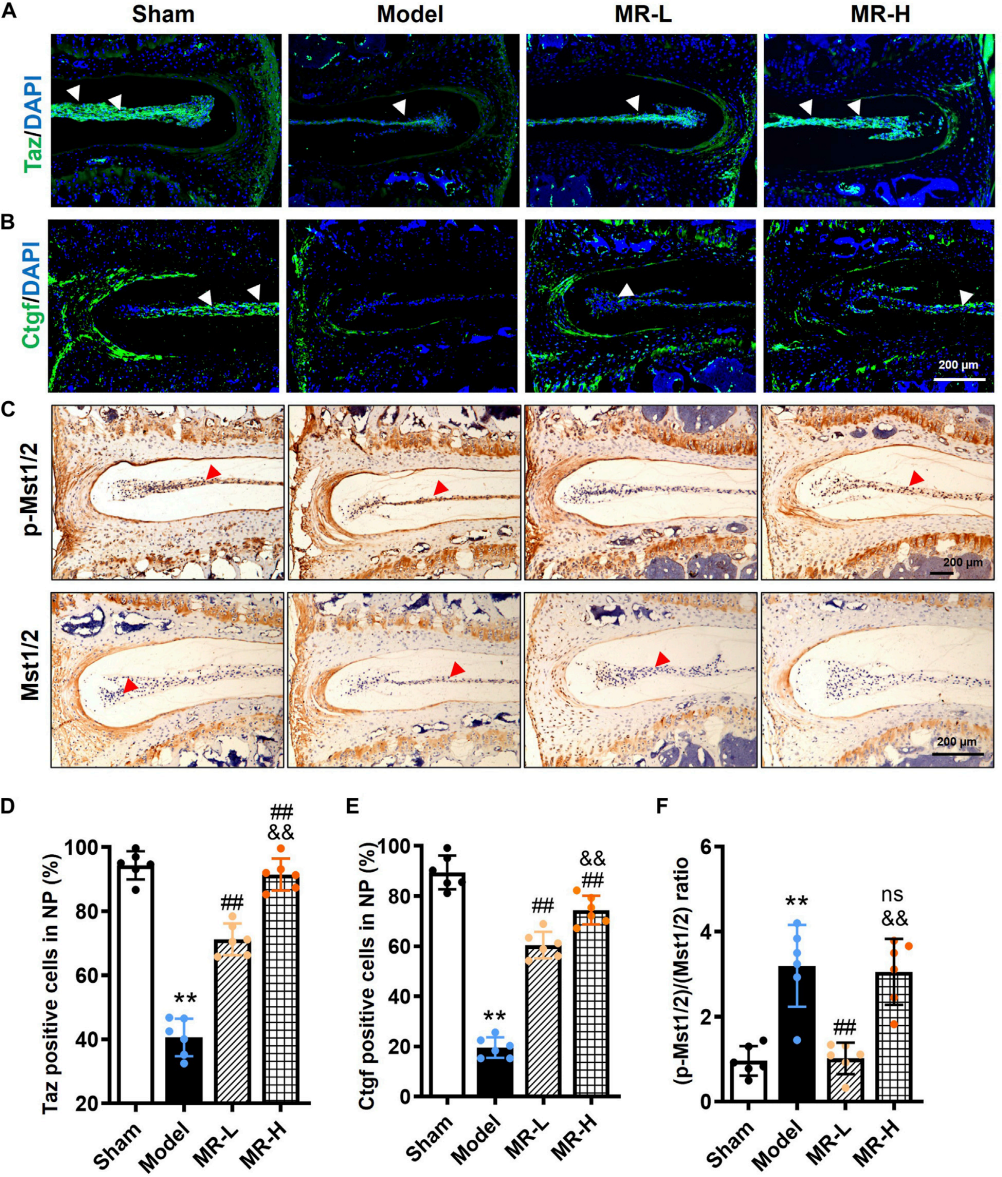
MR inhibits Hippo signaling of NP tissue in IVDD mice. **(A,B)** The IF staining results of Taz and Ctgf in NP tissues of mice. White arrowheads indicated positively-stained cells. **(C)** The IHC staining results of p-Mst1/2 and Mst1/2 in NP tissues of mice. Red arrowheads indicated positively-stained cells. **(D,E)** Quantification of Taz and Ctgf in **(A,B)**. **(F)** Quantification of the ratio of (p-Mst1/2)/(Mst1/2) in **(C)**. Data are expressed as mean ± SD, **p < 0.01 (vs. Sham group); #p < 0.05, ##p < 0.01 (vs. IVDD group), &&p < 0.01 (vs. MR-L group), *n* = 10 per group.

To further validate the potential pharmacological mechanism of MR in improving NP cell senescence *in vivo*, we determined Hippo signaling alterations in IVD tissues of MR-treated IVDD mice. IF data of Taz revealed that MR dose-dependently restored the reduction of Taz protein induced by LSI surgery in NP tissues of IVDD mice to levels observed in Sham mice (Figures 5A,B,D,E); however, no obvious Yap expression was observed in NP tissues (data not shown). Recent evidence has shown that recombinant Ctgf protein, a downstream target of Hippo signaling, could alleviate IVDD progression in rats (Ge et al., 2019). Similar to the expression pattern of Taz, we found that MR dose-dependently blocked Ctgf reduction in NP tissues of IVDD mice to levels comparable to those in Sham mice. Unexpectedly, we found that low-dose MR intervention significantly lowered the increased p-Mst1/2/Mst1/2 ratio by 68% in NP tissues of IVDD mice, whereas high-dose MR yielded no obvious effect (Figures 5C,F).

Furthermore, it is well-established that transcription factors Tead1-4 serve as downstream effectors of the Hippo pathway to regulate the expression of multiple genes involved in cell senescence (Xie et al., 2013). IHC analysis of Tead1-4 revealed that Tead1 was significantly decreased in the IVDD group but largely elevated in the MR-H group. Interestingly, although a non-significant decrease of Tead3 was observed in IVDD mice compared with Sham mice, MR treatment upregulated its expression dose-dependently (Supplementary Figures S3B,C). Besides, no expression of Tead2 and Tead4 was observed in IVD tissues (data not shown). Based on the above results, we speculated that Tead1 primarily mediates Hippo signaling in various IVD biological processes, and MR intervention simultaneously increases Tead1 and Tead3 to control Hippo signaling activity.

## Discussion

**FIGURE 6:**
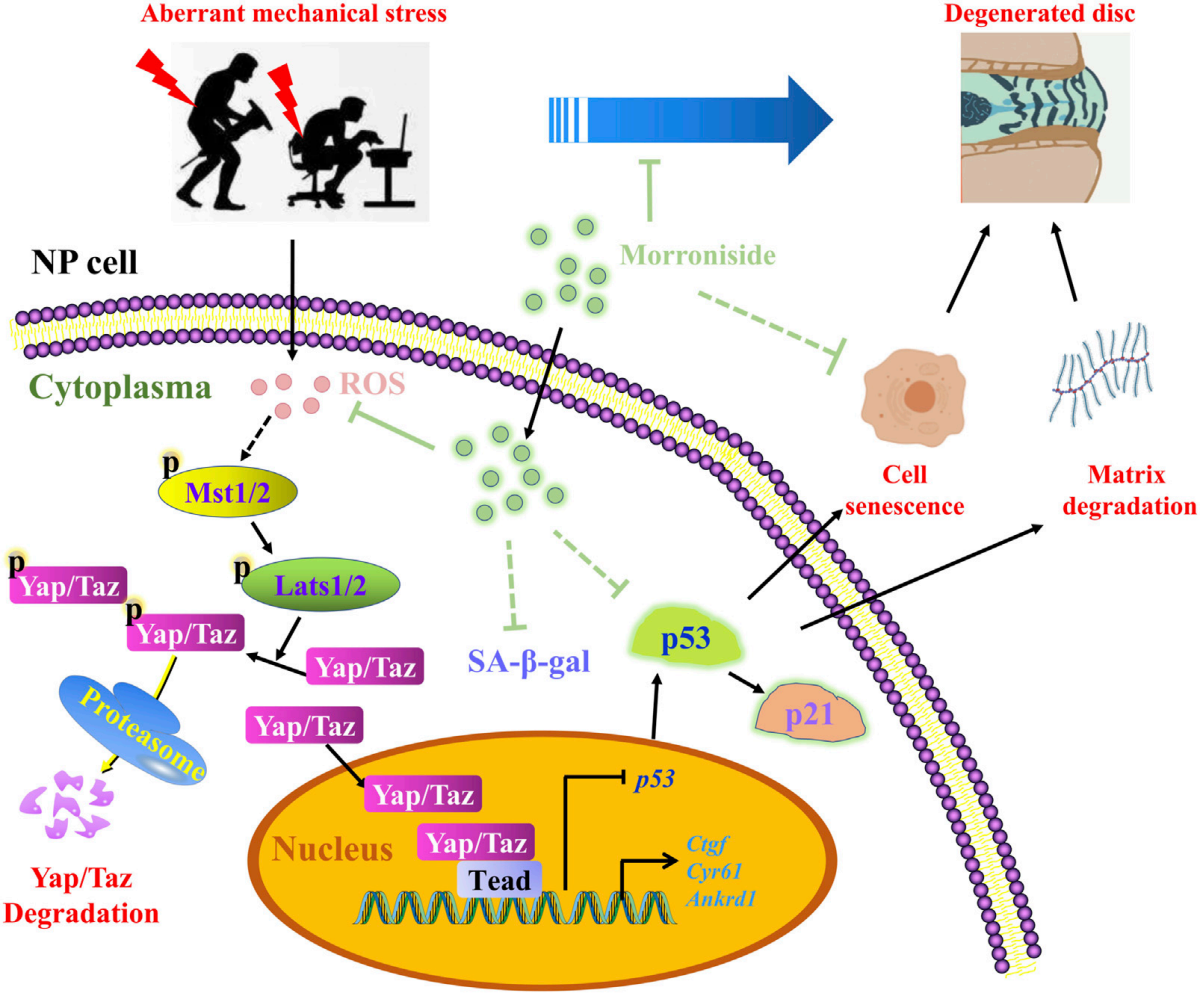
The schematic working model of MR-mediated protection against NP cell senescence and IVDD progression by targeting the ROS-Hippo-p53 pathway.

An increasing body of evidence suggests that CO-containing formulae (including Liuwei Dihuang formula) can effectively attenuate degenerative joint diseases, such as IVDD and osteoarthritis (Wang et al., 2018a; Yang et al., 2019; Zhang et al., 2019; Zhang et al., 2021c). A clinical trial (Identifier: NCT04108832) conducted in 2019 substantiated the efficacy of the Liuwei Dihuang formula against osteoarthritis. More importantly, our latest findings demonstrated that MR, a major iridoid glycoside derived from CO, could effectively attenuate cartilage degeneration and osteoarthritis development (Yu et al., 2021), comparable to the therapeutic effect of CO-containing formulae (Wang et al., 2018a; Yang et al., 2019; Zhang et al., 2019; Zhang et al., 2021c). Based on these findings, we hypothesized that MR plays a beneficial role against IVDD. In the present study, we utilized *in vitro* and *in vivo* models to assess the pharmacological effect of MR on IVDD progression. Our results showed that administration with MR alleviated the decline in intervertebral disc height, matrix degradation, and aggravation of IVDD progression. In this regard, it has been found that MR could suppress the NP cell senescent phenotype by abolishing the increase in p53 and p21 expression and SA-β-gal activity *in vivo* and *in vitro*. Further analysis of the underlying mechanism demonstrated that MR suppressed p53 mRNA and protein expression *via* inhibiting the ROS-Hippo pathway (Figure 6). Our study findings enhance our current understanding of the pharmacological effects of MR on IVDD development, corroborating that MR is a promising therapeutic agent for the treatment of IVDD.

Although the pathogenesis of IVDD has not been fully elucidated, it has been established that the accumulation of senescent NP cells plays an important role during IVDD progression (Kim et al., 2009; Jeong et al., 2014). Current evidence suggests that p53 activates the transcription and activity of multiple components participating in cellular senescence and organismal aging (Rufini et al., 2013). Using NP specimens from patients with degenerative disc disease, Kim *et al.* demonstrated p53 and p21 expression in senescent NP chondrocytes in all specimens, but p16 was expressed in a few NP chondrocytes in only two specimens, indicating the p53-p21 pathway rather than p16 pathway, plays an important role in NP cell senescence (Kim et al., 2008; Kim et al., 2009). Consistent with these results, we found a surge of senescent phenotype in the LSI surgery-induced mouse IVDD model and H_2_O_2_-induced rNP cell senescence model, as indicated by increased activity of SA-β-gal and enhanced expression of p53 and p21. Paradoxically, the expression of p16 protein was detected in the rNP cell line but not in mouse IVD tissues. Another study in human NP cells and mouse tail suspension (TS)-induced IVDD model yielded different observation results. Novais et al. (2019) reported that p16 expression affected IVDD pathogenesis by regulating matrix homeostasis and cell senescent phenotype without altering the onset of senescence, and disc-specific ablation of *p16* could attenuate IVDD progression by inhibiting oxidative stress and cell senescence (Che et al., 2020). We speculated that this discrepancy in p16 expression could be due to the heterogeneous origin of p16 antibodies and the methods used.

In addition, the elimination of these senescent cells has the potential to improve IVDD progression (Cherif et al., 2020; Novais et al., 2021; Shao et al., 2021). In recent years, much emphasis has been placed on using natural compounds such as quercetin and o-Vanillin to alleviate degenerative diseases, including IVDD (Li W et al., 2019; Cherif et al., 2020; Shao et al., 2021), with the key advantages of low toxicity, suggesting their great potential for clinical translation. Consistent with the literature (Wang et al., 2018b; Liu et al., 2020), we substantiated that MR, a natural iridoid glycoside in CO, significantly improved disc matrix homeostasis, inhibited NP senescent phenotype, and ameliorated disc degeneration in IVDD mice. Its anti-senescence effect was further verified in the H_2_O_2_-induced rNP cell senescence model. Our *in vivo* and *in vitro* results provided hitherto undocumented evidence of the role of natural low-toxic MR as a senolytic agent in IVDD therapy, which has huge prospects for clinical applications.

MR has been documented to possess anti-oxidation, anti-inflammation, and anti-apoptosis properties (Gao et al., 2020; Deng et al., 2021; Park et al., 2021). It can exert a neuroprotective role in neurodegenerative diseases by eliminating ROS to enhance the total antioxidant capacity of the rat cortex, thereby preventing nerve cells from H_2_O_2_-induced oxidation injury (Wang et al., 2009; Yao et al., 2009; Zhang et al., 2017c; Li B et al., 2019). Likewise, MR has been proven to protect lung activity against aging by maintaining the cell proliferative state and normal cell morphology and inhibiting apoptosis (Chen et al., 2014). Consistent with the literature, we have demonstrated that MR can protect against cartilage degeneration and osteoarthritis development by improving chondrocyte survival and matrix metabolism, as well as inhibiting chondrocyte apoptosis dose-dependently (Cheng et al., 2015; Yu et al., 2021). Herein, our data showed that MR not only scavenged ROS and improved H_2_O_2_-induced NP cell senescence but also ameliorated senescence of NP tissue of IVDD mice, suggesting that MR may act as an antioxidant to protect NP cell senescence and IVDD progression.

Accumulating studies have demonstrated that Hippo signaling plays an important role in regulating cellular senescence, which is strongly associated with aging-related diseases, particularly IVDD (Fu et al., 2019; Yang et al., 2021). Yap and Taz are well-recognized transcriptional coactivators of Hippo signaling. A study evaluating the biological behavior of rabbit IVD tissues under varying degrees of hydrostatic pressure showed that high-magnitude dynamic hydrostatic pressure disrupted ECM homeostasis in the NP and inner AF by enhancing Hippo signaling-mediated apoptosis, and further biochemical analysis showed that Taz knockdown increased p53 and p21 expression, leading to induction of cellular senescence, and this alteration was suppressed by further knockdown of p53, indicating that Taz negatively regulates the functions of p53 and attenuates p53-mediated cellular senescence (Miyajima et al., 2020). Similarly, acute disc injury-induced IVDD mice exhibited cytoplasmic translocation and inactivation of Yap; *Yap* knockdown by lentivirus sh*Yap* significantly induced NP cell senescence by increasing p53 and p21 protein expression levels (Zhang et al., 2018a; Zhang et al., 2018b). Consistent with the literature, we found aberrant activation of Hippo signaling in mouse IVDD model and H_2_O_2_-induced rNP cells. Moreover, MR treatment inhibited Hippo signaling in NP tissues of IVDD mice and H_2_O_2_-induced rNP cells. Noteworthy, the loss of function of Yap and Taz hampered MR-mediated protection against H_2_O_2_-induced NP cell senescence by blocking the inhibition of MR on p53 mRNA and protein expression and its transcriptional activity. However, deactivation of Hippo signaling by Yap/Taz inhibitor-1 did not influence p21 mRNA and protein expression. These data suggest that MR reduced p53 expression and NP senescent phenotype by targeting Hippo signaling. Another study demonstrated that p21 transcription could be regulated through either p53-dependent or p53-independent pathways (Lee et al., 2014). The above findings suggest that the inhibitory effect of MR on H_2_O_2_-induced p21 mRNA and protein expression in rNP cells is mediated in a ROS-Hippo-p53-independent manner.

As transcription coactivators in Hippo signaling, Taz and Yap have distinct and overlapping functions in various bioprocesses (Plouffe et al., 2018). Over the years, few studies have explored the role of Taz-mediated Hippo signaling in IVDD development and NP cell senescence. It has been established that Yap expression decreases with age in the IVD (Zhang et al., 2018b), and Yap inhibition induces senescence in NP cells (Zhang et al., 2018a; Croft et al., 2021). Intriguingly, another study pointed out that IL-6 could activate Yap and was involved in the degeneration of NP cells (Chen et al., 2019). In our present study, the expression of Yap and Taz was verified in rNP cell experiments, and only Taz was found in NP tissues of IVDD mice. Therefore, the roles of Yap/Taz in IVDD pathogenesis remain controversial, warranting further research. To further distinguish their functions in the onset and development of IVDD, it is necessary to comprehensively analyze the spinal phenotype of transgene mice with conditional deletion or overexpression of Yap/Taz driven by Aggrecan-Cre^ERT2^ (cKO) in the future.

Besides, several limitations were present in this study. Our present results showed that MR supplementation could dose-dependently restore the expression of critical downstream proteins of Hippo signaling (Taz, Ctgf, and Tead1/3) in IVDD mice to levels observed in Sham mice, which in turn suppresses the p53 pathway and NP senescent phenotype. However, the histological NP senescent phenotype (SA-β-gal activity as well as p53 and p21 expression) and the matrix (Aggrecan) expression did not display a better therapeutic effect associated with high-dose MR on NP senescence and matrix degradation. It is unclear whether these alterations result from the inability of high-dose MR on dephosphorylation of Mst1/2. Thus, the roles of the Mst1/2 kinase-independent of the Hippo signaling pathway on NP senescence need further elucidation. Moreover, we speculate that MR may, directly and indirectly, target NP to treat IVDD. Our results substantiated that systematic administration of MR could directly inhibit NP cell senescent phenotype *in vivo* and *in vitro*, thereby ameliorating IVDD progression. However, MR intervention may also affect other tissues in the body to secret cytokines and chemokines to influence NP senescence. Therefore, MR might affect the secretory phenotype in tissues other than IVDs and exert indirect effects, which needs to be further confirmed by local intervention with MR within IVD tissue. Indeed, the specific mechanism underlying the regulatory role of MR on p21 expression remains obscure and needs further clarification.

In summary, our study demonstrates the accumulation of senescent NP cells in a mouse model of IVDD and H_2_O_2_-treated rNP cells. Besides, MR could effectively attenuate NP cell senescence to alleviate IVDD progression by targeting the ROS-Hippo-p53 pathway. The above data suggest a novel insight that MR could be a promising therapeutic agent in the treatment of IVDD.

## Data Availability

The original contributions presented in the study are included in the article/Supplementary Material, further inquiries can be directed to the corresponding authors.

## Supplementary material

SUPPLEMENTARY FIGURE S1 Quantitative analysis of MR using the HPLC method. The authentication result of MR was provided by the Manster TCM Co., Ltd., (Sichuan, China). The HPLC analysis was carried out on a Shimatsu LC-20A HPLC system with a MinXi Tech CG-C18 5mic column (250 mm × 4.6 mm) at 40°C. The mobile phase was 0.1% formic acid in deionized water and acetonitrile at a flow rate of 0.8 ml/min. The gradient elution program was as follows: 0–20 min, 10% acetonitrile; 20–40 min, 10%–60% acetonitrile. The sample volume was 10 μl.

SUPPLEMENTARY FIGURE S2 Effects of MR and H_2_O_2_ treatments on rNP cells. rNP cells were treated with specified concentrations of H_2_O_2_ for 2 h. Western blot results showed that H_2_O_2_ induced expression of senescence-related proteins p53 and p21. **(B)** qPCR assay results showed that expression of p53 and p21 mRNA levels were increased in H_2_O_2_-treated rNP cells. **(C)** SA-β-gal staining results showed that SA-β-gal-positive cells were increased after treatment with H_2_O_2_. **(D)** Western blot results revealed the effects of H_2_O_2_ treatment on the phosphorylation status of Mst1/2, Lats1/2, and Yap/Taz. **(E)** Western blot results indicated that H_2_O_2_ dose-dependently reduced Taz protein expression. **(F)** qPCR assay results showed that supplementation with H_2_O_2_ induced the expression of ankrd1, ctgf, and cyr61 mRNA levels. **(G)** Results of intracellular ROS levels. Values are expressed as mean ± SD. ^*^ *P <* 0.05, ^**^ *P <* 0.01 compared with Vehicle-treated cells. ^##^ *P <* 0.01 compared with H_2_O_2_-treated cells.

SUPPLEMENTARY FIGURE S3 Effects of MR on the viability of H_2_O_2_-treated rNP cells and Tead1/3 expression in NP tissues of IVDD mice. CCK-8 assay. rNP cells were pretreated with the indicated concentration of MR for 2 h, followed by 200 μM H_2_O_2_ for another 2 h. The proliferation of rNP cells was determined 24 h after H_2_O_2_ incubation. **(B)** The IHC staining results of Tead1 and Tead3 in NP tissues. Red arrowheads indicate positive staining cells. **(C)** Quantification of Tead1 and Tead3 positive cells in **(B)**. Data are expressed as the mean ± SD, ^**^ *P <* 0.01 (vs. Sham group); ^ns^ *P* > 0.05 (vs. IVDD group), ^#^ *P <* 0.05, ^##^ *P <* 0.01 (vs. IVDD group), ^ns^ *P* > 0.05 (vs. IVDD group), ^&&^ *P* < 0.01 (vs. MR-L group).
