# Supplementary figures and images for: Morroniside attenuates nucleus pulposus cell senescence to alleviate intervertebral disc degeneration via inhibiting ROS-Hippo-p53 pathway
Source: Front Pharmacol. 2022 Sep 16;13:942435. doi: 10.3389/fphar.2022.942435 (PMC9524229; doi:10.3389/fphar.2022.942435)

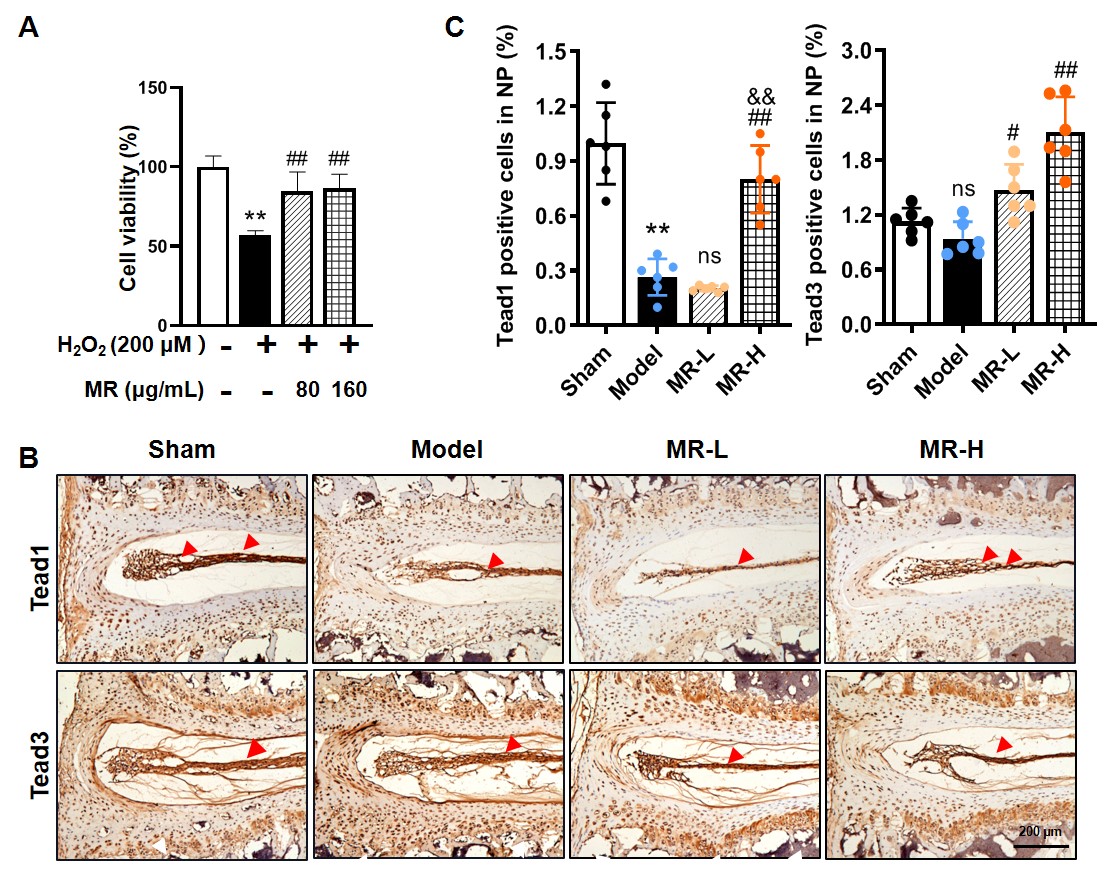

Supplement: Supplementary file 1 [file Image3.jpg]

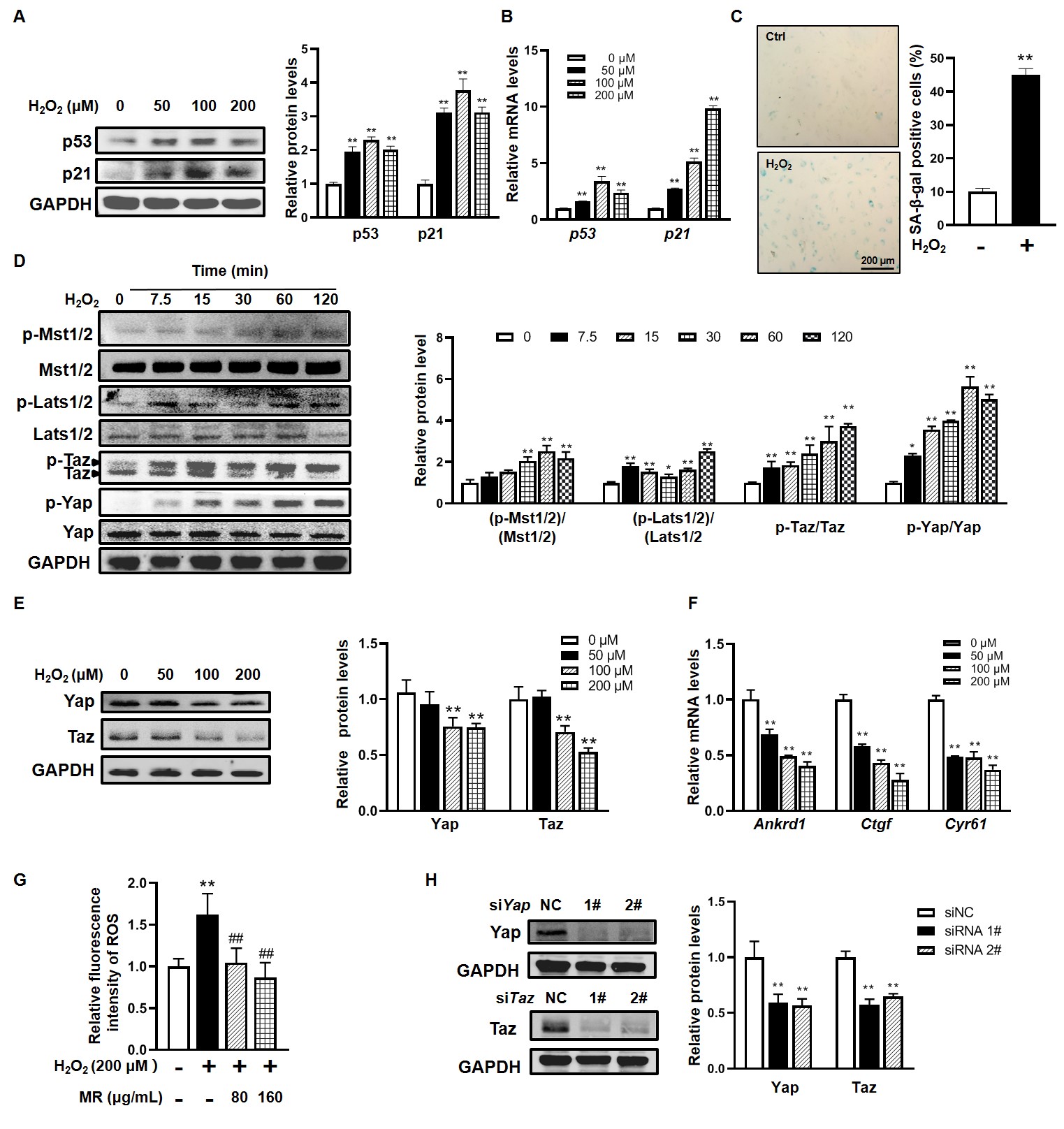

Supplement: Supplementary file 2 [file Image2.jpg]

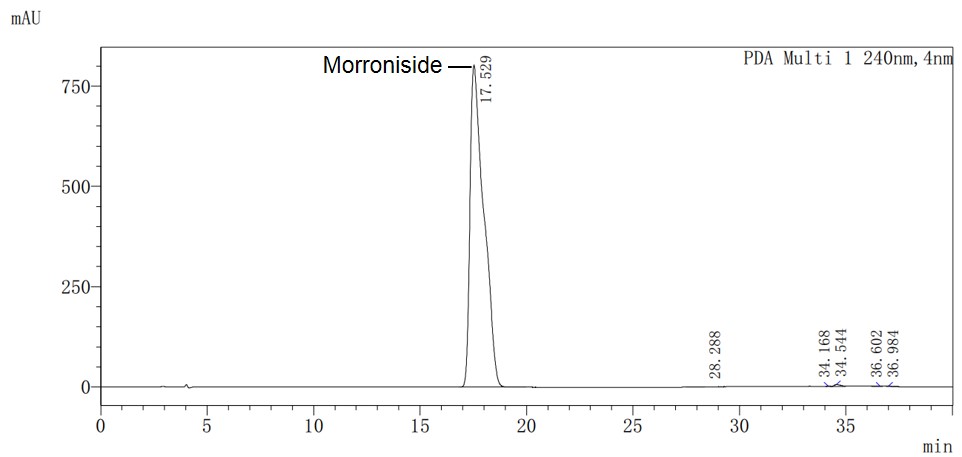

Supplement: Supplementary file 3 [file Image1.jpg]
